# Supplementary material for: Assessing the Impacts of Creating Active Schools on Organisational Culture for Physical Activity
Source: Int J Environ Res Public Health. 2022 Dec 16;19(24):16950. doi: 10.3390/ijerph192416950 (PMC9778943; doi:10.3390/ijerph192416950)
Supplement: Supplementary file 1 [file ijerph-19-16950-s001.zip › Questionnaire S1 - Creating Active Schools Organisational Change Questionnaire.pdf]

Introduction

Thank-you for your interest in this survey - it will take around 30 minutes to complete. We will also contact you in 6 and every 12 months after that to complete the same questionnaire. Before you decide whether or not to take part, please read the research information sheet in this link. By completing the questionnaire (paper or electronic version), you are providing consent for the University of Bradford to use your responses for research purposes.

(electronic version, Please click on the 'next page' button to begin - If you do not want to answer a question you can miss it out.

If you don't have time to finish the questionnaire now, **you can save your answers and come back at any time by clicking the 'save' button at the bottom of any page.**

Name	
Role	
Number of years working in education	
School	
Date	

Please complete all questions below by circling the answer that best describes the current picture within your school. There are no right or wrong answers and your responses will not be shared with anyone else.

Whole school culture and ethos

1. Our school has a **clear strategy and ethos to support pupils to be physically active** both within and beyond the school day

strongly disagree   disagree   neutral   agree   strongly agree

2. Our school **policies clearly detail how, where and when inclusive physical activity opportunities are offered for all pupils** in order to achieve at least 30 minutes of in-school physical activity

strongly disagree   disagree   neutral   agree   strongly agree

3. Our school **policies prioritise the need for training to support staff in providing high-quality physical activity** opportunities for all pupils

strongly disagree   disagree   neutral   agree   strongly agree

4. Our school **improvement plan identifies what outcomes we need to achieve** to influence children's physical activity behaviour

strongly disagree   disagree   neutral   agree   strongly agree

5. Our school **improvement plan identifies how we will achieve the outcomes** relating to children's physical activity behaviour

strongly disagree   disagree   neutral   agree   strongly agree

6. Our school **monitors and evaluates** our activities related to supporting children to be physically active (e.g. records what is delivered, how it is delivered, and levels of engagement from children, what's gone well, and what challenges have occurred)

strongly disagree   disagree   neutral   agree   strongly agree

7. Our school **uses the evidence from our own monitoring and evaluation to inform** how we support children to be active

strongly disagree   disagree   neutral   agree   strongly agree

8. Our school **uses research evidence** (publications, research advice) and national policy and guidance (obesity plan, PESSP guidance) **to inform** how we support children to be active

strongly disagree *disagree* *neutral* *agree* *strongly agree*

9. Our school has a **designated whole-school physical activity lead that encompasses more than P.E. and School Sport.**

strongly disagree *disagree* *neutral* *agree* *strongly agree*

Communication

1. Our **external communications** (e.g. newsletters, social media, prospectus, website) clearly show that physical activity is a core priority.

strongly disagree *disagree* *neutral* *agree* *strongly agree*

2. Our **internal communications** (e.g. staff meeting agendas, staff appraisals, assemblies, displays within our school) clearly show that physical activity is a core priority.

strongly disagree *disagree* *neutral* *agree* *strongly agree*

Physical Environments

1. Our **school hall** is suitable (e.g. markings, size) to facilitate a wide range of physical activity opportunities for all children

strongly disagree *disagree* *neutral* *agree* *strongly agree*

2. Our **school corridors** are suitable to facilitate a wide range of physical activity opportunities for all children

strongly disagree *disagree* *neutral* *agree* *strongly agree*

3. Our **school's hard standing areas** (outdoor spaces) are suitable to facilitate a wide range of physical activity opportunities for all children

strongly disagree *disagree* *neutral* *agree* *strongly agree*

4. Our school's **green spaces** are suitable (e.g. markings, size) to facilitate a wide range of physical activity opportunities for all children

strongly disagree *disagree* *neutral* *agree* *strongly agree*

5. Our school ensures that **children can access outdoor areas** for physical activity in all weather

strongly disagree *disagree* *neutral* *agree* *strongly agree*

6. Our school has **access to spaces and facilities beyond the school premises** (recreation field, playgrounds, sports halls) that can facilitate a wide range of physical activity opportunities for all children

strongly disagree *disagree* *neutral* *agree* *strongly agree*

7. Our school has **regular access to a high-quality swimming pool** to promote swimming for all pupils

strongly disagree *disagree* *neutral* *agree* *strongly agree*

Stakeholders

Senior Leaders (includes head teacher, assistant head) in your school……..

1. have the **knowledge** to facilitate the delivery of whole-school physical activity (C)

strongly disagree *disagree* *neutral* *agree* *strongly agree*

2. have the **skills** to develop a whole-school approach to physical activity (C)

strongly disagree *disagree* *neutral* *agree* *strongly agree*

3. are **visible in their support and encouragement of school staff** to deliver physical activity opportunities (O)

strongly disagree *disagree* *neutral* *agree* *strongly agree*

4. **believe our school can provide sufficient physical activity opportunities for all pupils** that can be feasibly implemented (O)

strongly disagree *disagree* *neutral* *agree* *strongly agree*

5. believe engagement in physical activity can **positively influence pupils' physical well-being** (M)

strongly disagree *disagree* *neutral* *agree* *strongly agree*

6. believe that engagement in physical activity can **positively influence pupils' mental well-being** (M)

strongly disagree *disagree* *neutral* *agree* *strongly agree*

7. believe that engagement in physical activity can **positively influence pupils' academic performance**(M)

strongly disagree *disagree* *neutral* *agree* *strongly agree*

8. believe that incorporating physical activity across the school day is a **worthwhile use of their time** (M)

strongly disagree *disagree* *neutral* *agree* *strongly agree*

Teachers and wider school staff (e.g teaching assistants) in your school….

1. **positively support each other** to deliver a whole-school approach to physical activity (O)

strongly disagree *disagree* *neutral* *agree* *strongly agree*

2. believe that engagement in physical activity can **positively influence pupil's physical well-being** (M)

strongly disagree *disagree* *neutral* *agree* *strongly agree*

3. believe that engagement in physical activity can **positively influence pupils' mental well-being** (M)

strongly disagree *disagree* *neutral* *agree* *strongly agree*

4. believe that engagement in physical activity can **positively influence pupils' academic performance** (M)

strongly disagree *disagree* *neutral* *agree* *strongly agree*

5. believe that incorporating physical activity across the school day is a **worthwhile use of their time** (M)

strongly disagree *disagree* *neutral* *agree* *strongly agree*

6. **demonstrate dedication and ambition to incorporate physical activity** across the school day (C)

strongly disagree *disagree* *neutral* *agree* *strongly agree*

7. are **provided with high-quality, evidence-based training** to support their knowledge and skills to provide high quality physical activity for all pupils (C)

strongly disagree *disagree* *neutral* *agree* *strongly agree*

Children in our school…...

1. are given the **opportunity to influence decisions** on the resources and opportunities provided for physical activity (e.g., pupil council) (O)

strongly disagree *disagree* *neutral* *agree* *strongly agree*

2. **support each other** to be physically active (O)

strongly disagree *disagree* *neutral* *agree* *strongly agree*

3. are given the **opportunity to train to be physical activity volunteers and champions** (O)

strongly disagree *disagree* *neutral* *agree* *strongly agree*

Parents of the children in our school…...

1. are given the **opportunity to influence decisions** relating to resources and opportunities provided for physical activity (O)

strongly disagree *disagree* *neutral* *agree* *strongly agree*

2. are given the **opportunity and relevant training to volunteer to assist with in-school physical activity opportunities** such as break times, events, before/after school activities.(O)

strongly disagree *disagree* *neutral* *agree* *strongly agree*

Community stakeholders who work with our school…...

1. **share best practice and support the development and delivery of a wide variety of school-based physical activity initiatives** (O)

strongly disagree *disagree* *neutral* *agree* *strongly agree*

Opportunities

Physical activity during academic (non PE) lessons

1. The majority of teachers know how to **integrate** physical activity into academic lessons (C)

strongly disagree *disagree* *neutral* *agree* *strongly agree*

2. The majority of teachers **have the skills to integrate physical activity** into the delivery of academic content (e.g. physically active learning). (C)

strongly disagree *disagree* *neutral* *agree* *strongly agree*

3. The majority of teachers have **been provided with high-quality, evidence-based training** to deliver physically active academic lessons (C)

strongly disagree *disagree* *neutral* *agree* *strongly agree*

4. The majority of teachers **incorporate physical activity into their curriculum lessons on most days** (O)

strongly disagree *disagree* *neutral* *agree* *strongly agree*

5. The majority of teachers **incorporate classroom movement breaks** (e.g., Brain Breaks, Energizers, GoNoodle, moving about the classroom, stretching, etc.) **into lessons on most days.** (O)

strongly disagree *disagree* *neutral* *agree* *strongly agree*

6. The majority of teachers have **been provided with sufficient high quality teaching resources** to support the delivery of physical activity in academic lessons (O)

strongly disagree *disagree* *neutral* *agree* *strongly agree*

7. Our **school's classrooms are suitable to facilitate appropriate physical activity** opportunities for all children (O)

strongly disagree *disagree* *neutral* *agree* *strongly agree*

8. The majority of teachers have the **knowledge and skills to deliver physically active academic lessons outside of the classroom** (C)

strongly disagree *disagree* *neutral* *agree* *strongly agree*

Physical activity during Physical Education

1. The majority of teachers have the **knowledge** to deliver high quality physical education lessons (C)

strongly disagree *disagree* *neutral* *agree* *strongly agree*

2. The majority of teachers have the **skills** to deliver high-quality physical education lessons (C)

strongly disagree *disagree* *neutral* *agree* *strongly agree*

3. The majority of teachers have **been provided with high-quality, evidence-based training** to deliver high-quality physical education. (C)

strongly disagree *disagree* *neutral* *agree* *strongly agree*

4. Our **school's indoor space for PE is always prioritised for delivery of the PE curriculum during the day,** and not a split use space where other classes are held or lunch is served (O)

strongly disagree *disagree* *neutral* *agree* *strongly agree*

5. **PE lessons are well resourced with a wide range of high-quality equipment** sufficient to engage all children simultaneously (O)

strongly disagree *disagree* *neutral* *agree* *strongly agree*

6. Our school provides **two hours of high-quality PE per week** for all pupils (O)

strongly disagree *disagree* *neutral* *agree* *strongly agree*

Physical activity during break/ lunch

1. The majority of break/lunch supervisors have the **knowledge** to effectively support children's physical activity (C)

strongly disagree *disagree* *neutral* *agree* *strongly agree*

2. The majority of break/lunch supervisors have the **skills** to facilitate high-quality physical activity. (C)

strongly disagree *disagree* *neutral* *agree* *strongly agree*

3. The majority of break/lunch supervisors have been **provided with high-quality, evidence-based training** to facilitate physical activity during break/lunch (C)

strongly disagree *disagree* *neutral* *agree* *strongly agree*

4. Pupils have **access to a wide range of high-quality physical activity equipment** during break/lunch (O)

strongly disagree *disagree* *neutral* *agree* *strongly agree*

5. Our school has **sufficient space for all pupils to be physically active during break/lunch** (O)

strongly disagree *disagree* *neutral* *agree* *strongly agree*

6. Our school **provides sufficient opportunities for pupils to be active** during wet break and lunch times. (O)

strongly disagree *disagree* *neutral* *agree* *strongly agree*

Physical activity during events/ visits

1. Our school **regularly provides free or low-cost opportunities for children to visit external sites** (e.g. local green spaces, sports centres) that encourage and promote physical activity (O)

strongly disagree *disagree* *neutral* *agree* *strongly agree*

2. Our school **provides opportunities for all children to take part in intra/inter school physical activity events** that focus on and reward participation (O)

strongly disagree *disagree* *neutral* *agree* *strongly agree*

The following opportunities relate to physical activity during before/ after school clubs

1. The majority of before/ after school session leaders have the **knowledge** to effectively support children's physical activity (C)

strongly disagree *disagree* *neutral* *agree* *strongly agree*

2. The majority of before/ after school session leaders have the **skills** to facilitate high-quality physical activity. (C)

strongly disagree *disagree* *neutral* *agree* *strongly agree*

3. The majority of before/ after school session leaders have been **provided with high-quality, evidence-based training** to facilitate physical activity within their activities. (C)

strongly disagree *disagree* *neutral* *agree* *strongly agree*

4. Our school **provides a wide variety of supervised before/after school non-traditional physical activities** (e.g. walking, gardening, orienteering) (O)

strongly disagree *disagree* *neutral* *agree* *strongly agree*

5. Our school provides a **wide variety of supervised before/after school traditional and competitive sporting activities** (e.g. football, athletics, tennis, netball) (O)

strongly disagree *disagree* *neutral* *agree* *strongly agree*

6. Our school **supports all pupils to regularly access our before/after school physical activities** (O)

strongly disagree *disagree* *neutral* *agree* *strongly agree*

Physical activity during the commute to/from school

1. Our school **regularly uses strategies** (e.g., walk to school week, celebration of active travel) to encourage pupils to use active transportation (O)

strongly disagree *disagree* *neutral* *agree* *strongly agree*

2. Our school provides **regular training to support all pupils** to actively travel to school (cycling proficiency, balance bikes, scooters, green cross code) (C)

strongly disagree *disagree* *neutral* *agree* *strongly agree*

3. Our school **active travel plan is comprehensive and up to date** (N/A)

strongly disagree *disagree* *neutral* *agree* *strongly agree*

4. Our school **provides families with information about designated Safe Routes to School** (C)

strongly disagree *disagree* *neutral* *agree* *strongly agree*

5. Our school **provides access to secure and sufficient storage of bikes and scooters** (O)

strongly disagree *disagree* *neutral* *agree* *strongly agree*

6. Our school **provides access to a walking bus or similar active transportation programme** (e.g. park and stride) (O)

strongly disagree *disagree* *neutral* *agree* *strongly agree*

7. Our **school staff are role models** for active travel (O)

strongly disagree *disagree* *neutral* *agree* *strongly agree*

Physical activity beyond school time

1. Our school **supports** (e.g. physically active homework) **parents and families to facilitate physical activity for their children** outside of school (O)

strongly disagree *disagree* *neutral* *agree* *strongly agree*

2. Our **regular communications with parents** (e.g. newsletters, social media, parent portal, parent evenings) **encourage and provide clear signposting to opportunities for children to be active** within (e.g. before/after school clubs) and beyond the school (e.g. local clubs, greenspaces and community organisations) (O)

strongly disagree *disagree* *neutral* *agree* *strongly agree*

3. Our school **provides parents with regular opportunities to take part in physical activity with their child** at home and within school (O)

strongly disagree *disagree* *neutral* *agree* *strongly agree*
