# Supplementary figures and images for: Assessing the Impacts of Creating Active Schools on Organisational Culture for Physical Activity
Source: Int J Environ Res Public Health. 2022 Dec 16;19(24):16950. doi: 10.3390/ijerph192416950 (PMC9778943; doi:10.3390/ijerph192416950)

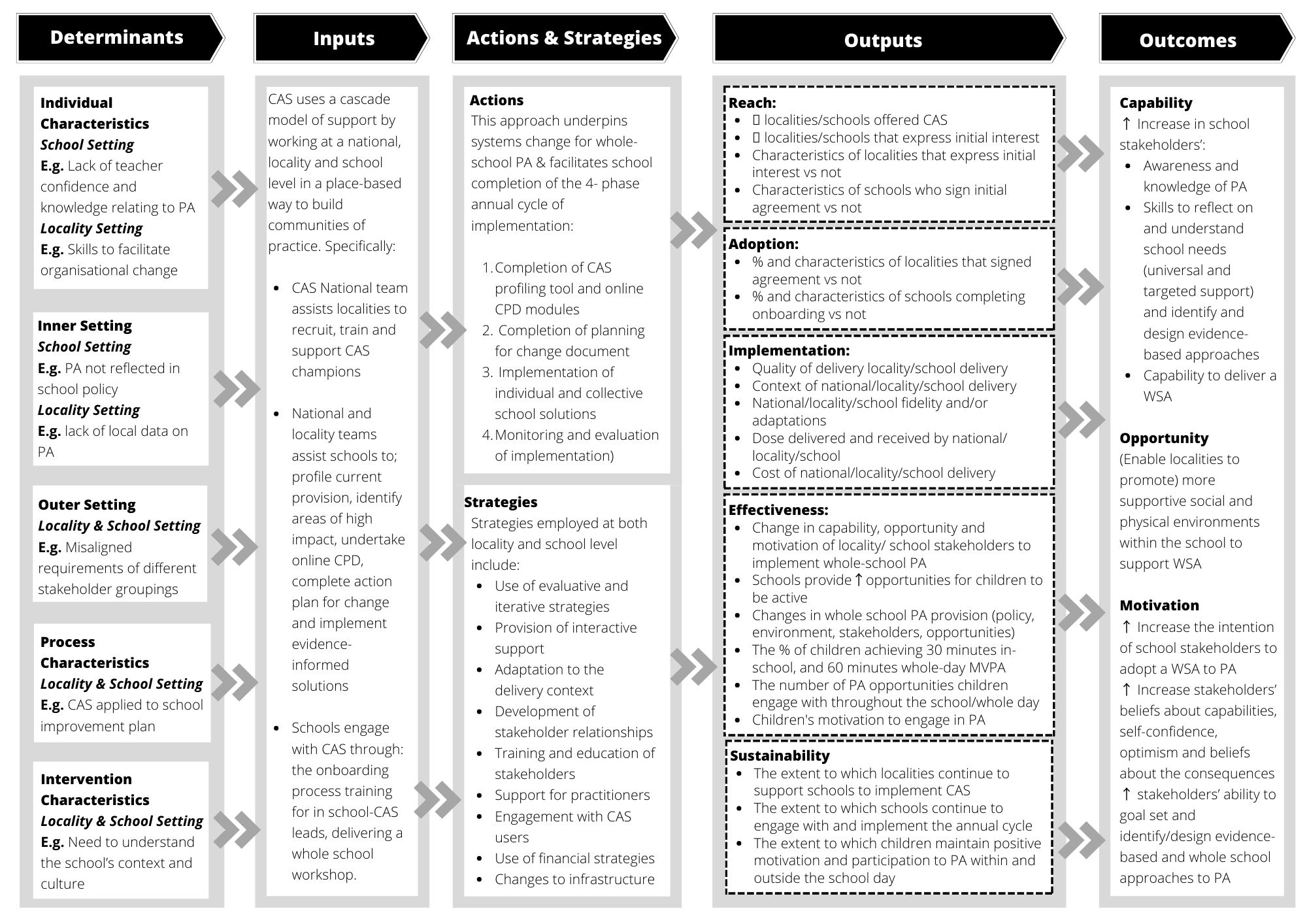

Supplement: Supplementary file 1 [file ijerph-19-16950-s001.zip › Figure S1- CAS Logic Model.jpg]

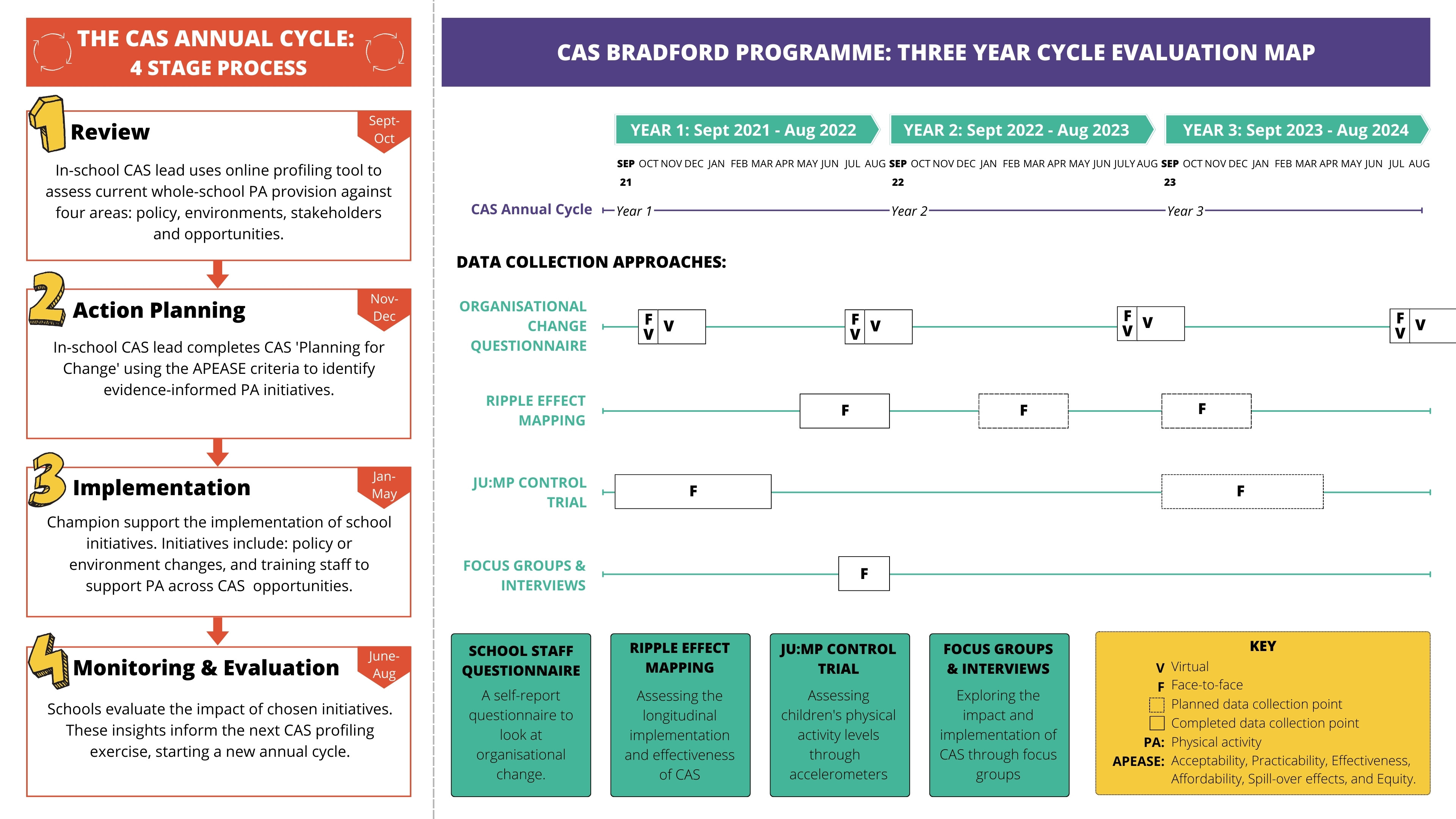

Supplement: Supplementary file 1 [file ijerph-19-16950-s001.zip › Figure S2- CAS Evaluation Plan.jpg]
